# A Cardiac Cell Outgrowth Assay for Evaluating Drug Compounds Using a Cardiac Spheroid-on-a-Chip Device

**DOI:** 10.3390/bioengineering5020036

**Published:** 2018-05-04

**Authors:** Jonas Christoffersson, Florian Meier, Henning Kempf, Kristin Schwanke, Michelle Coffee, Mario Beilmann, Robert Zweigerdt, Carl-Fredrik Mandenius

**Affiliations:** 1 Division of Biotechnology, Department of Physics, Chemistry and Biology (IFM), Linköping University, 58183 Linköping, Sweden; jonas.christoffersson@liu.se; 2 Boehringer Ingelheim Pharma GmbH and Co. KG, Nonclinical Drug Safety Germany, D-88397 Biberach an der Riss, Germany; florian.meier@boehringer-ingelheim.com (F.M.); mario.beilmann@boehringer-ingelheim.com (M.B.); 3 Leibniz Research Laboratories for Biotechnology and Artificial Organs (LEBAO), Hannover Medical School, Carl-Neuberg-Str. 1, 30625 Hannover, Germany; Kempf.Henning@mh-hannover.de (H.K.); Schwanke.Kristin@mh-hannover.de (K.S.); Coffee.Michelle@mh-hannover.de (M.C.)

**Keywords:** 3D cell culture, microfluidics, organ-on-a-chip, cardiac spheroids, cardiomyocytes, induced pluripotent stem cells (iPSCs), drug screening

## Abstract

Three-dimensional (3D) models with cells arranged in clusters or spheroids have emerged as valuable tools to improve physiological relevance in drug screening. One of the challenges with cells cultured in 3D, especially for high-throughput applications, is to quickly and non-invasively assess the cellular state in vitro. In this article, we show that the number of cells growing out from human induced pluripotent stem cell (hiPSC)-derived cardiac spheroids can be quantified to serve as an indicator of a drug’s effect on spheroids captured in a microfluidic device. Combining this spheroid-on-a-chip with confocal high content imaging reveals easily accessible, quantitative outgrowth data. We found that effects on outgrowing cell numbers correlate to the concentrations of relevant pharmacological compounds and could thus serve as a practical readout to monitor drug effects. Here, we demonstrate the potential of this semi-high-throughput “cardiac cell outgrowth assay” with six compounds at three concentrations applied to spheroids for 48 h. The image-based readout complements end-point assays or may be used as a non-invasive assay for quality control during long-term culture.

## 1. Introduction

The recent development of perfused three-dimensional (3D) cell culture models, or organs-on-chip, offers the possibility to investigate biological responses of chemicals and pharmaceuticals in a model that better mimics the in vivo cell environment than conventional two-dimensional culture models [1,2]. Therefore, results from such assays are believed to increase the predictivity of drug effects on human tissue such as efficacy and toxicity. Advanced in vitro assays may thus better predict harmful or ineffective chemicals before they enter the long and expensive drug development process. Common approaches to create a 3D cell environment are to embed the cells in a hydrogel matrix such as collagen [3] or Matrigel [4], or to let the cells aggregate into cell spheroids [5,6,7]. A critical challenge for both 2D and 3D-based assays is to examine the impact of compounds on the target cells without substantial interference. For continuous non-invasive assaying, several methods have been developed to analyze the supernatant of the cell culture medium to reveal the cellular state in sequential off-line monitoring of biomarkers [8,9]. Furthermore, for cardiac cells, standard methods include the recording of beating frequency and electrocardiographic recording using microelectrode arrays which can be performed non-invasively [10,11]. However, recording videos of cells is time consuming, and electrocardiography is mostly performed on 2D cardiomyocytes. Analysis of cell growth and morphology have previously been reported for several cell types such as neurites in the neuronal network formation assay and endothelial cells in the wound healing assay [12,13]. However, with respect to cardiac assays, the outgrowth of cells has been described as a naturally occurring process which, in primary tissue, may result from cardiac progenitor cells [14]. Compared to conventional static conditions, dynamic cell cultures have been shown to have positive effects on several cell types [15,16,17] and also to support functional outputs of cardiac aggregates [18].

In this article, we combine recent progress on the derivation of human pluripotent stem cell-cardiomyocytes (CMs), their use for engineering cardiac tissue including spheroids, and in microfluidics technology for developing novel drug testing assays. The approach is based on quantifying the number of cells growing out from cardiac spheroids within a defined time and area, by combining solvent controls versus exposure to six compounds at three concentrations. Non-invasive, microscopy-based assessment showed substantial effects of doxorubicin, endothelin-1 (both decreasing cell outgrowth), and amiodarone (support cell outgrowth). To objectively determine the cell outgrowth around the spheroids, cell nuclei were stained and counted using a high content imaging system which also revealed the effect of phenylephrine (increased outgrowth). Comparisons were also made between static and dynamic cultures, and between cardiac spheroids derived from two different human induced pluripotent stem cell (hiPSC) lines, both confirming the drug- and dose-dependent effects.

With the challenges of analyzing 3D cell spheroids in mind, this novel approach for investigating the effect of chemicals or drug compounds could be used as a compliment to invasive end-point assays or as a non-invasive quality control tool used during long-term cultures.

## 2. Materials and Methods

### 2.1. Cell Lines and Preparation of Cardiac Spheroids

**Figure 1:**
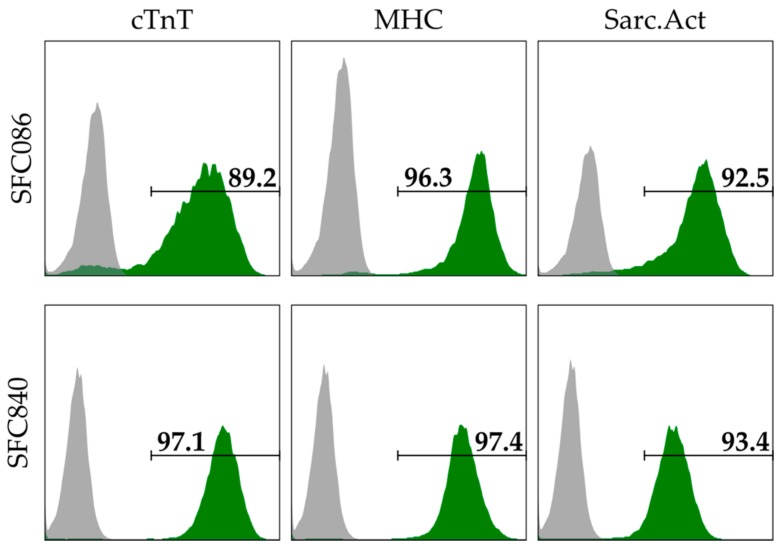
Flow cytometry-based assessment of cardiomyocyte (CM) content using immunofluorescent stains specific to cardiac troponin T (cTnT), pan-myosin heavy chain (MHC), and sarcomeric actinin (Sarc.Act). Depending on the marker, the CM content of the SFC086 cell line and the SFC840 cell line was ~89–96% and ~93–97% respectively. CM content was assessed at day 10–14 after induction of differentiation, i.e., when the cells were used for spheroid formation.

Cardiac spheroids, each consisting of approximately 2500 cells (~250 µm in diameter) were generated as follows. The human induced pluripotent stem cell lines (hiPSC) SFC086-03-01 and SFC840-03-01 (referred to as SFC086 and SFC840, respectively, and derived by the StemBANCC initiative [19] http://stembancc.org/; received from the Human Biomaterials Resource Centre, University of Birmingham (http://www.birmingham.ac.uk/facilities/hbrc)) were cultured and differentiated by recently established protocols in suspension culture [20,21,22] to achieve a cardiomyocyte (CM) content of ~90% (SFC086) and >90% (SFC840), respectively (Figure 1). Briefly, cells were dissociated using collagenase IV (Life Technologies) and subsequently resuspended in medium consisting of Iscove’s modified Dulbecco’s medium with GlutaMAX™ (Life Technologies/ Thermo Fisher Scientific, Waltham, MA, USA) supplemented with 20% fetal bovine serum, 0.2 mM l-glutamine, 0.1 mM b-mercaptoethanol, 1% non-essential amino acids (*v*/*v*), 1 mg/mL penicillin, and 1 U/mL streptomycin and 10 µM Rho-associated coiled-coil kinase (ROCK) inhibitor Y-27632. Cells were then reseeded into Statarrays© MCA96-16.224-PSLA Low Attachment Surface plates (300MICRONS, Karlsruhe, Germany) with the concentration of 2500 cells per microcavity, in 300 µL of the same medium to form spheroids within 48 h. Spheroids were recovered by pipetting gently, transferred to RB+ medium consisting of RPMI1640 supplemented with B27 with insulin (Life Technologies) and used for experiments within 5–10 days after generation.

### 2.2. Flow Cytometry and Immunofluorescent Staining

For intracellular staining, 1.5 × 10^5^ differentiated cells were fixed/permeabilized according to manufacturer’s instructions (Fix&Perm-kit by An der Grub, Thermo Fisher Scientific, Waltham, MA, USA). Antibodies specific to cardiac troponin T (1:200, clone 13-11, Thermo Fisher Scientific), sarcomeric actinin (α-ACTININ; 1:800, clone EA-53, Sigma-Aldrich, St. Louis, MO, USA), myosin heavy chain (MHC; 1:25, Hybridoma Bank, Iowa City, IA, USA; 1:2000, clone NOQ7.5.4D, Sigma-Aldrich), NKX2.5 (1:200; clone H-114; Santa Cruz, California, CA, USA), and respective isotype controls (Dako, Agilent, Santa Clara, CA, USA) were detected using appropriate Cy3-/Cy5-conjugated antibodies (1:200; Jackson Immunoresearch Laboratories, West Grove, PA, USA). After washing, signals were detected using Cy3-labeled donkey anti-mouse IgM (1:200; Jackson Immunoresearch Laboratories) on the Accuri C6 flow cytometer (BD Biosciences, Franklin Lakes, NJ, USA). Data were analyzed using FlowJo (Treestar, FlowJo, LLC, Ashland, OR, USA).

Plated aggregates/outgrowing cells were fixed with 4% paraformaldehyde, 15 min, room temperature (RT). After blocking by Tris-buffered saline (5% donkey serum, 0.25% Triton X-100), cells were incubated with primary/secondary antibodies listed above, respectively. Nuclei were DAPI-stained, and samples were analyzed using the Axio Observer A1 (Zeiss, Jena, Germany) or a DM IRB/TCS SP2 confocal microscope system (Leica, Wetzlar, Germany).

### 2.3. Microfluidic System and Experimental Procedure for Compound Testing

Microfluidic channel slides with dimensions 17 × 3.8 × 0.4 mm (length × width × height) (Ibidi µSlide VI^0.4^, untreated) were coated to facilitate cell attachment by adding 30 µL laminin (100 µg/mL) to each channel for 1 h at room temperature, followed by washing with PBS and cell culture medium before seeding the cell spheroids. Cardiac spheroids were infused by adding 30 µL with approximately 20 spheroids to one of the wells and rapidly removing 20 µL cell culture medium from the opposite well. The spheroids were allowed to attach to the surface of the channel at 37 °C and 5% CO_2_. After 16 h incubation, the cell culture medium in each channel was removed and substituted to 90 µL compound supplemented cell culture medium or control (0.25% DMSO) before mounting the slide on a motorized rocking table for continuous alternating perfusion of cell culture medium with a frequency of 0.5 Hz at 37 °C and 5% CO_2_. (see also Supplementary Figure S1). Applied compounds Doxorubicin, endothelin-1, acetylsalicylic acid, isoproterenol, phenylephrine, and amiodarone were all purchased from Sigma and diluted in a final concentration of 0.25% DMSO in cell culture medium. The concentrations used were 0.04 nM, 1.11 nM, and 10 nM of endothelin-1, and 0.04 µM, 1.11 µM, and 10 µM for all other compounds. After 48 h of treatment, spheroids were washed with PBS, fixed in 4% PFA for 1 h at room temperature, and the cell nuclei were then stained with Hoechst 33342.

### 2.4. Image Processing and Nuclei Quantification

Images of the spheroids were captured by a camera (Canon PowerShot A640, Canon Tokyo, Japan) attached to a phase microscope (Zeiss Axiovert 40C, Zeiss) or, for quantification of the number of nuclei around the spheroids, by a high content imaging system (Opera Phenix, Perkin Elmer, Waltham, MA, USA). Images were taken with a 5× objective in confocal mode. Image analysis was performed with the Harmony (Perkin Elmer) software. The number of nuclei was normalized to the area of analysis around each spheroid to account for variations in size (see Supplementary Figure S2). Statistical difference between control samples of the SFC086 and SFC840 cell lines, both run in triplicates and counting the number of nuclei around at least 18 spheroids in each channel, was determined by a two-pair *t*-test at a level of α = 0.05. Statistical difference between control samples and the number of nuclei around at least six spheroids for each compound treatment was determined by a *z*-test at a level of α = 0.05.

## 3. Results

**Figure 2:**
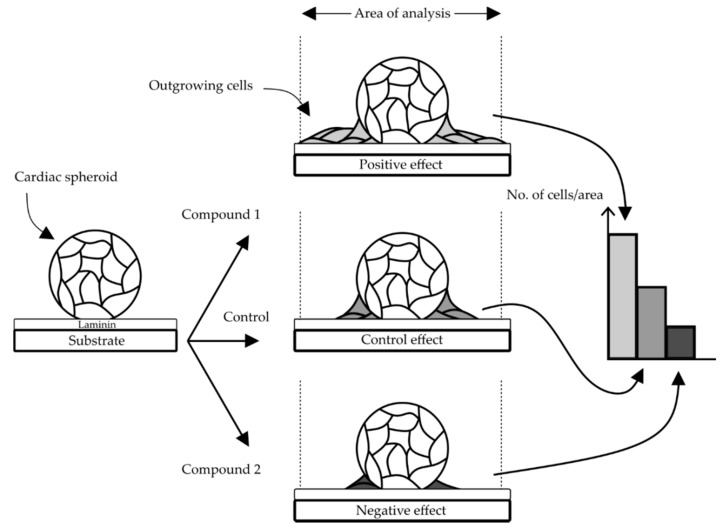
Concept of the proposed cardiac cell outgrowth assay. An outgrowth of cells from spheroids over time was observed on laminin coated surfaces (control) and the number of cells within a defined area should be possible to quantify and potentially reveal an increased (positive) or decreased (negative) effect depending on the compound.

During routine culture of cardiac spheroids on laminin coated surfaces, we observed that some cardiac cells tended to grow out from the aggregates and attach to the surrounding surface. From this observation we hypothesized that this effect could be exploited for developing a cell-based assay able to assess effects of a drug (Figure 2). Specifically, the assay should be based on the assumption that exposing cardiac spheroids to compounds would affect the number of surrounding cells, and by that, assess both negative (decreased cell number) and positive (increased cell number) effects on the particular cell type’s ability to proliferate or migrate, in relation to the compound.

### 3.1. Design of the Cell Outgrowth Assay

**Figure 3:**
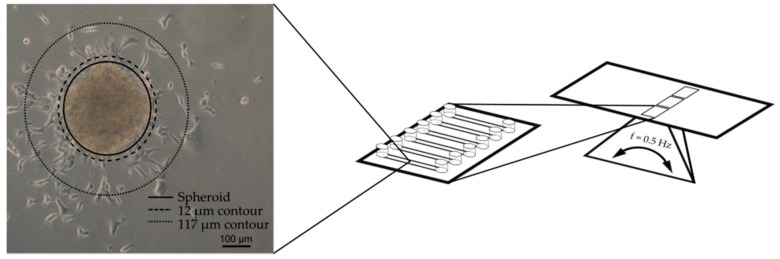
Cardiac spheroids were seeded in fluidic channels on a microscope slide-format and fixed on a rocking platform to drive the perfusion through the device by gravity. The number of nuclei within the area confined by the 12 µm and 117 µm contours were counted by high content imaging.

**Figure 4:**
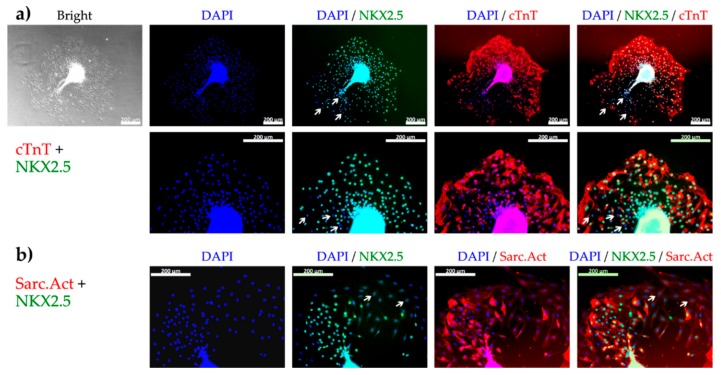
The presence of cardiomyocytes (CM) among cells growing out from the spheroids 48 h after plating determined by fluorescence microscopy of CM specific markers. (**a**) Bright field image of cell outgrowth from a spheroid and subsequent fluorescence microscopy showing nuclei (DAPI, blue), early CM specific transcription factor (NKX2.5, green), and cardiac troponin T (cTnT, red), at 10× (top) and 20× (bottom) magnification. (**b**) Fluorescence microscopy showing nuclei (blue), NKX2.5 (green), and sarcomeric actinin (Sarc.Act, red) at 20× magnification. Most of the cells were positive for both NKX2.5 and cTnT or NKX2.5 and Sarc.Act revealing their CM phenotype. White arrows show NKX2.5 negative nuclei indicating the presence of non-cardiac cells. Scale bars represent 200 µm.

To realize the outgrowth assay in an appropriate microfluidic format, the design had to accommodate several critical functions. These included a contained fluidized space for the cell spheroids, a geometric format suitable for high content screening, a device with several parallel cell culture areas to allow a semi-high throughput procedure, and an expedient way to perfuse the device with cell culture medium. These criteria were matched by an existing commercial device on a microscope slide format (Figure 3). The device consisted of six parallel channels with adjacent reservoirs that could be fixed on a motorized rocker to drive the perfusion of cell culture medium by gravity. By coating the channels with laminin, it was possible to capture the cardiac spheroids in the device for subsequent imaging by light microscopy and confocal imaging. The cardiac spheroids in each image were identified by automatic thresholding by the high content imaging software. A contour was applied 12 µm (5 pixels) outside the boundary of the spheroids to create a space from the dense cardiac spheroids. A second contour was applied 117 µm (50 pixels) from the spheroids to define the area of analysis within the two contours. Because several areas were spotted where closely neighboring spheroids occasionally fused into larger clusters, the area of analysis was made to ensure distinctly separated clusters. The labelling of cardiac specific markers on cells growing out from the spheroids confirmed the predominant presence of CMs (Figure 4) and a relatively small portion of non-cardiac cells (white arrows, Figure 4) in line with the flow cytometry data, suggesting high content of CMs in the differentiated cell suspension used for spheroid formation.

### 3.2. Verification of the Assay Performance with Six Compounds

Six compounds, all with either potential negative, negligible, or positive effect on cardiac cell growth, were chosen for verifying the assay performance (Table 1). The compounds included doxorubicin, a drug compound used in chemotherapy with well-documented toxic effects on heart cells [23]; endothelin-1, an established vasoconstrictor and hypertrophy inducer [24]; acetylsalicylic acid, an analgesic and anti-inflammatory compound [25]; isoproterenol, a β-adrenergic receptor agonist and positive inotrope [26]; phenylephrine, a α-adrenergic receptor agonist and vasoconstrictor [27]; and amiodarone, a K^+^ channel blocker and antiarrhythmic agent [28]. These compounds were selected due to their diverse but well-established effects on cardiac cells (see Table 1). We assume that these drugs showed low and similar interactions with the laminin-coated surfaces of the device, although it cannot be excluded that minor outgrowth effects might have occurred in the experiments.

**Figure 5:**
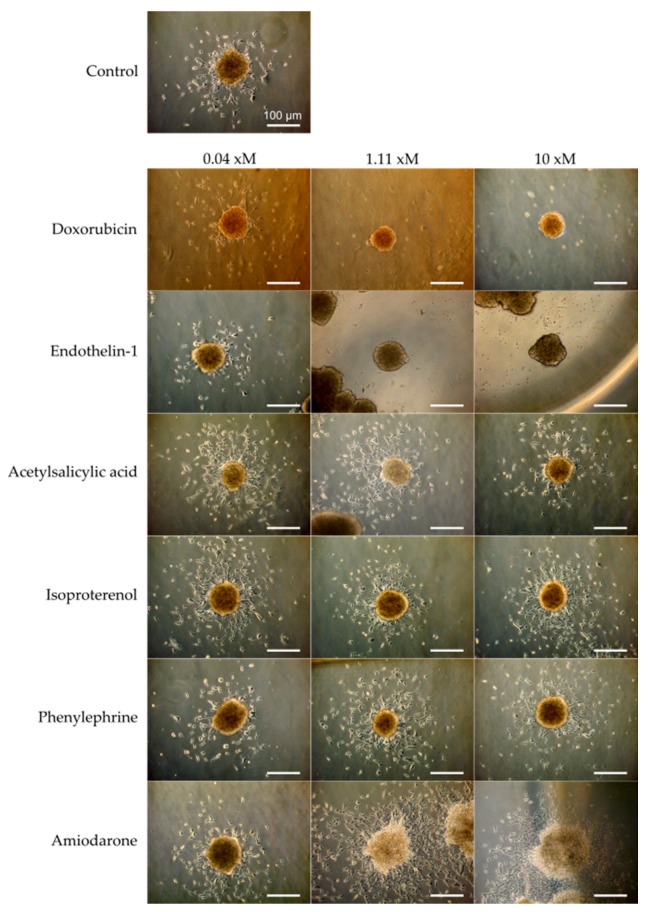
Micrographs of cardiac spheroids (SFC086) showing the outgrowth of cells from the aggregates and the effect of six drug compounds at three concentrations after 48 h on the number of cells around the spheroids. For endothelin-1, x = n (nM). For all other compounds, x = µ (µM). Scale bar represents 100 µm.

**Figure 6:**
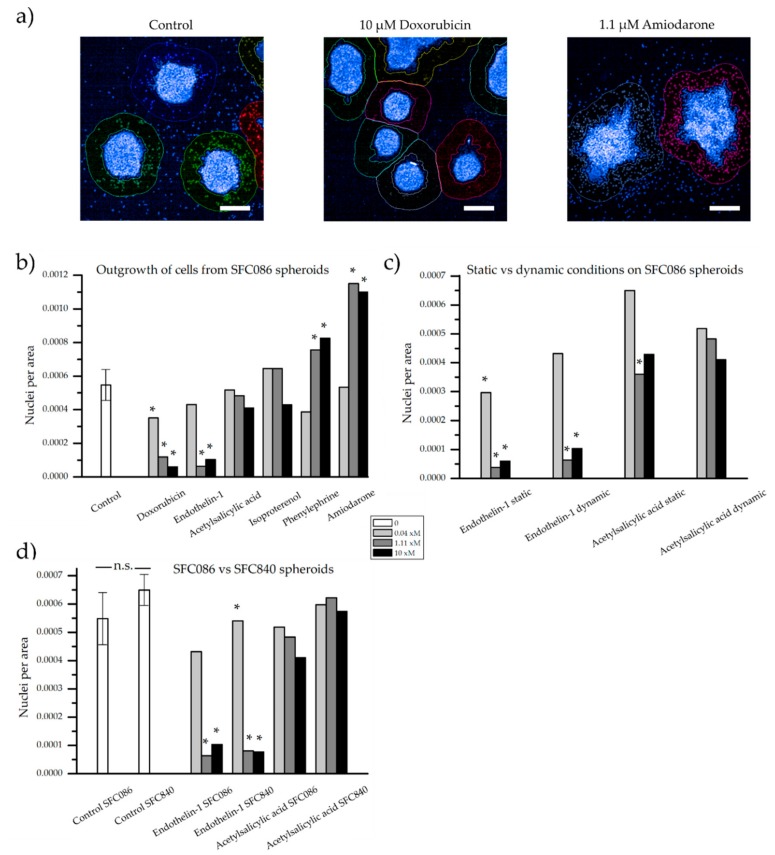
Quantification of the number of nuclei visible outside a defined area of the spheroids. (**a**) Examples of the applied contours around spheroids stained with Hoechst 33342 and captured by high content imaging. Number of nuclei per area of analysis from (**b**) the SFC086 cell line under dynamic conditions exposed to six compounds at three concentrations, (**c**) the SFC086 cell line at static and dynamic conditions exposed to two compounds, and (**d**) the SFC086 cell line compared to the SFC840 cell line when exposed to two compounds. Control samples are presented as mean ± standard deviation of three experiments, * denotes a significant difference of the sample from the dynamic control. No significance (n.s.) was observed between the two control samples (SFC086 and SFC840). For endothelin-1, x = n (nM). For all other compounds, x = µ (µM). Scalebar represents 200 µm.

After 48 h of treatment, cells growing out from the cardiac spheroid were first visualized by phase contrast microscopy (Figure 5). All spheroids were still contractile at the time of fixation, but the rate was not quantified during these experiments. In control samples, cells were found inhabiting the laminin coated surface surrounding the spheroids. A drastic decrease of cell outgrowth was apparent in the presence of doxorubicin at 1.11 µM and 10 µM, and of endothelin-1 at 1.11 nM and 10 nM. The opposite effect was observed with amiodarone at 1.11 µM and 10 µM inducing increase cell outgrowth. In the presence of acetylsalicylic acid, isoproterenol, and phenylephrine, minor effects compared to control sample were observed. For more accurate quantification, cell nuclei stained with Hoescht 33342 were assessed and counted within the area of analysis by the Opera high content imaging system (Figure 6a). As presented in Figure 6b, the effect of the highest doses of doxorubicin, endothelin-1, and amiodarone, which were readily indicated by more qualitative phase contrast monitoring, was confirmed. Furthermore, a significant difference compared to control samples was now detected already at 0.04 µM of doxorubicin, and at 1.11 µM and 10 µM of phenylephrine.

Indications of more sensitive cells under static conditions, when compared to the dynamic control, were noted, as fewer nuclei per area surrounded the spheroids when treated with enothelin-1 from 0.04 nM and in acetylsalicylic acid at 1.11 µM (Figure 6c). However, any clear differences were neither found for endothelin-1 nor for acetylsalicylic acid but could be of interest to study in a larger experiment. Furthermore, as compound-exposed cardiac spheroids at static conditions were compared to control samples at dynamic conditions, any distinct conclusions would be difficult to draw from these experiments. Nevertheless, similar patterns, with fewer cells around endothelin-1 exposed spheroids compared to acetylsalicylic acid exposed spheroids, were observed at both dynamic and static conditions.

Finally, we compared cardiac spheroids derived from the hiPSC lines SFC086 versus SFC840 (cardiomyocyte content of ~90–95% for both lines) in our assay. Results were highly consistent between both lines (Figure 6d), suggesting cell line independent reproducibility and general relevance of the method.

## 4. Discussion

The results demonstrated a pragmatic quantitative strategy to assess drug effect on cardiac cells at the interplay of 3D/2D and static/dynamic culture. For this to occur, a method for capturing cell spheroids in a fluidic device followed by continuous dynamic culture was established. The setup is robust and simple to use even for newcomers in the field of microfluidic cell culture users. We show experimental conditions enabling to use the “cardiac cell outgrowth from 3D spheroids” as a quantitative measurement to examine significant drug effects. It may be argued that the assay not completely mirrors 3D conditions since the assay partly takes place outside the spheroids and should therefore be considered a “2.5D” assay. However, significant drug interactions representative for the assay readout occur inside the spheroids.

The method is fast and enables continuous non-invasive cell monitoring by standard light microscopy as a readout, which deems compatible with automated high content imaging and screening. Subsequently, the approach can be used as an end-point assay in combination with a potentially more accurately quantifiable fluorescence-based analysis of counterstained nuclei.

To validate assays’ utility for monitoring drug effects, we decided to use five compounds with known impact on the heart (doxorubicin, endothelin-1, isoproterenol, phenylephrine, and amiodarone), and one control drug that may not affect cardiomyocyte function (acetylsalicylic acid), all tested at three concentrations. The decreased number of cells around spheroids exposed to doxorubicin can be attributed to the compound’s established cytotoxicity, which has previously been shown to cause cardiac cell death [23]. Endothelin-1 is associated with hypertrophic responses on cardiac cells such as increased cell size and elevated levels of B-type natriuretic peptide (BNP) expression [19], myofibrillar disarray [29], and has been shown to increase the survival of doxorubicin treated rat cardiomyocytes in short-term (24 h) [30]. These data suggest that cytotoxicity may not be predominantly responsible for the decreased cell outgrowth in spheroids exposed to endothelin-1. However, the reported myofibrillar disarray due to ET-1 [29] may suggest an overall detrimental impact of the drug on cytoskeletal function, thereby reducing cardiomyocytes motility and thus limiting their outgrowth in our assay. Whilst further mechanistic analysis is required, these observations highlight the sensitivity of our assay since the effect was readily observed at a relative low ET-1 dose of 1.11 nM.

No significant effect could be observed at any concentration of isoproterenol. The beta-adrenergic agonist isoproterenol is therapeutically applied to increase the heart rate in patients suffering from bradycardia (slow heart rate). In our approach, we have solely focused on differences in cell morphological outputs and not measured functional attributes such as the beating rate, but notably the general experimental setup is compatible with beating rate assessment.

Equivalent to endothelin-1, phenylephrine is a known vasoconstrictor [27]. Interestingly, an opposite effect of these two drugs, that is an increased outgrowth upon phenylephrine administration versus outgrowth suppression by ET-1, was observed. Phenylephrine mimics epinephrine which both have been shown to promote cell growth of cardiac fibroblasts and increasing the cell size of neonatal cardiomyocytes [31].

The increased number of cells around spheroids treated with amiodarone is less predictive from published literature. Previous studies on hiPSC-derived cardiomyocytes showed contradicting effects on cells’ beating rate in response to amiodarone ranging from no effect at up to 10 µM [32] to decreased beating at up to 100 µM [33] and increased frequency at up to 100 µM [34]; this heterogeneity may be attributed to cell- or assay-dependent effects. However, we did not find published data on CM proliferation or motility in response to amiodarone administration, which has apparently not been identified by other assays before.

Besides the use in advanced drug testing assays, human pluripotent stem cell-derived CMs have also been considered in regenerative medicine [35]. One central problem in that field is the low implantation efficiency and pure cell distribution in the heart post cell administration [36,37,38]. Although we have not performed animal studies here, it is tempting to speculate that those drugs, that support CMs outgrowth in our in vitro assay, may also support proliferation and better distribution of transplanted cells in cardiac tissue, thereby facilitating heart repair.

The CMs used for spheroid generation were derived by well-defined procedures in suspension culture [20,21] using chemical Wnt pathway modulators to ensure lineage-specific differentiation and high CMs content [22,39]. It is worth noting that such CMs represent an immature phenotype with respect to their gene and protein expression pattern and physiological properties [40,41]. Compared to their functional counterparts in the adult heart, which are known to be cell cycle arrested and non-proliferative, human pluripotent stem cell derived CMs maintain a relative high proliferation potential and expression of proliferation-associated markers such a Ki-67 [40]. This indicates that the “CMs outgrowth phenotype” monitored in our assay is likely attributed to both cardiomyocyte proliferation as well as motility. Although our assay covers the result of both such effects and was mainly developed as a primary screen to identify compounds and respective concentrations, a detailed analysis to distinguish between these phenomena is necessary to reveal molecular mechanism(s) of specific compounds. Thus, our focus here is on developing a novel assay strategy for measuring drug effects which are not covered by other applied assays and which provide valuable information on novel, potentially unexpected effects of respective drugs. In that way, the outgrowth assay endpoints have a clear relevance for drug assessment.

Importantly, the data in our study were closely recapitulated by using CMs derived from two independent hiPSC lines, strongly supporting robustness and general (rather than cell line dependent) relevance of the method and its results.

Finally, aggregates of cardiomyocytes cultured under dynamic conditions have been reported to grow in size, contain more nuclei, and display increased contraction forces compared to static cultures. However, we did not observe any (potentially expected) differences between static and dynamic culture conditions in our assay, although a clearly observable gravity-induced medium flow was achieved via the microfluidic design on the rocker-device. Further studies for dynamic studies may implement a higher shear rate/stress compared to our approach (mean value approximately 4.5 dyn/cm^2^). This can be achieved, for example, by connecting an external pump or by increasing the height of the well to create a higher velocity of the fluid through the channel. This may be a critical issue if the size of the microchannels are smaller than in those in this report. On the other hand, it remains open how such experimental flow conditions will compare e.g., to tissue perfusion in an organ such as the heart.

## 5. Conclusions

In this paper, the number of cells surrounding cardiac spheroids attached to the surfaces of perfused microfluidic channels was used as a quantitative measurement of the effect of different compounds. By staining the cell nuclei with a fluorescent marker, it was possible to induce both a decrease (doxorubicin and endothelin-1) and an increase (phenylephrine and amiodarone) in number of cells surrounding the spheroids.

Based on these findings, we suggest that this measurement procedure is useful as an assay for evaluating drug molecules in a 3D environment. However, the assay should preferably be performed as a complement to already existing end-point assays for providing supportive data. This could for example include beating rate.

The assay is carried out in a spheroid-on-a-chip device. A few caveats should be mentioned with this format. Drug molecules of varying hydrophobicity may be affected by surface properties of the fabrication materials of device. Also, the conditions for the cells grown out from the spheroids in the microfluidic channel may not be fully representative for the 3D model. These could be parameters to be further evaluated in validation of the assay.

The spheroid chip format is cost-effective, requires short training before practice, and can easily be scaled-up to higher throughout for routine laboratory work. This makes the spheroid-on-a-chip a convenient complementary assay tool for drug development.

## Figures and Tables

**Table 1:** The compounds used in these experiments and their expected effect on cell outgrowth.

Compound	Type	Mechanism	Expected Effect (−/\/+) ^1^
Doxorubicin	Chemotherapeutic	DNA intercalator	−
Endothelin-1	Vasoconstrictor	Hypertrophy inducer	−
Acetylsalicylic acid	Analgesic	Anti-inflammation	\
Isoproterenol	Positive inotrope	β-adrenergic receptor agonist	+
Phenylephrine	Vasoconstrictor	α-adrenergic receptor agonist	+
Amiodarone	Antiarrhythmic agent	K^+^ channel blocker	+

^1^ The expected effect was either negative (−), negligible (), or positive (+) with regard to cell number increase in the area of analyses.

## Supplementary Materials

The following are available online at http://www.mdpi.com/2306-5354/5/2/36/s1, Figure S1: Schematics showing the dynamic condition of the spheroid-on-a-chip device on the rocker setup. The frequency of the rocker was 0.5 Hertz, or 30 alternating cycles per minute, Figure S2: Depiction of the different areas detected by the high content imaging system. The blue area represents the attached spheroid. The red circle shows the area surrounding the spheroid which was excluded from the analysis to avoid any counting of cells which were still located in the spheroid. The green area represents the analyzed area for analysis. Hoechst 33342 positive nuclei in this area were counted and normalized to the area of the green circle.
