# Supplementary material for: A Cardiac Cell Outgrowth Assay for Evaluating Drug Compounds Using a Cardiac Spheroid-on-a-Chip Device
Source: Bioengineering (Basel). 2018 May 4;5(2):36. doi: 10.3390/bioengineering5020036 (PMC6027518; doi:10.3390/bioengineering5020036)
Supplement: Supplementary file 1 [file bioengineering-05-00036-s001.pdf]

Supplementary Materials

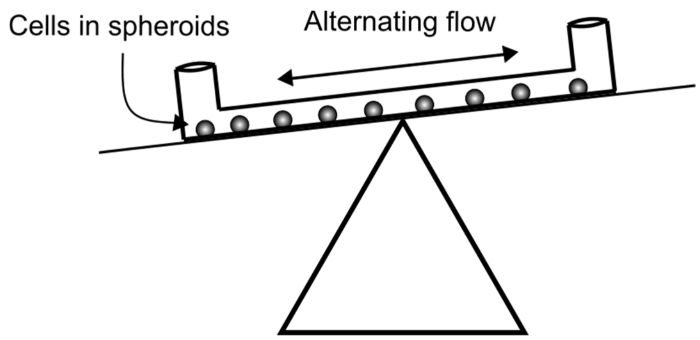

Figure S1. Schematics showing the dynamic condition of the spheroid-on-a-chip device on the rocker setup. The frequency of the rocker was 0.5 Hertz, or 30 alternating cycles per minute.

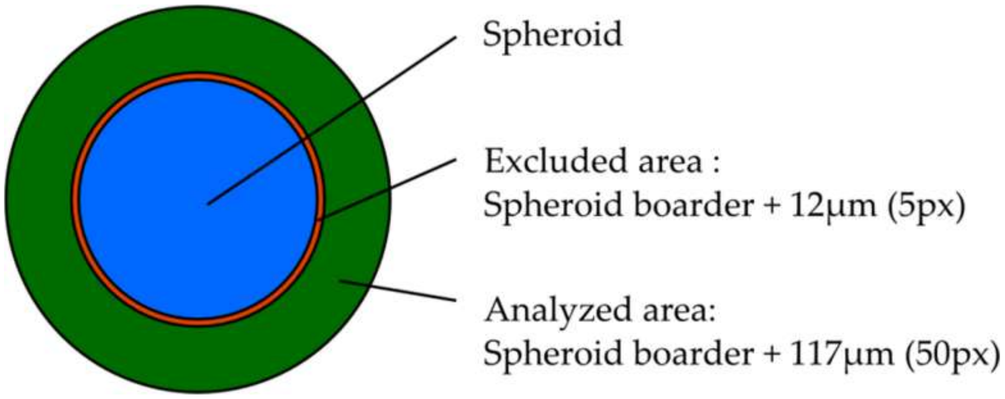

Figure S2. Depiction of the different areas detected by the high content imaging system. The blue area represents the attached spheroid. The red circle shows the area surrounding the spheroid which was excluded from the analysis to avoid any counting of cells which were still located in the spheroid. The green area represents the analyzed area for analysis. Hoechst 33342 positive nuclei in this area were counted and normalized to the area of the green circle.
